# Radiation-induced Lung Injury CT Features: Early Non-Small Cell Lung Cancer SBRT Prognosticators

**DOI:** 10.2174/0115734056388161250320055401

**Published:** 2025-04-09

**Authors:** Fang Wang, Lingling Wang, Hong Yang, Yujin Xu, Haitao Jiang

**Affiliations:** 1 Department of Radiology, Zhejiang Cancer Hospital, Hangzhou, Zhejiang, China; 2 Wenzhou Medical University, Department of Radiology, Wenzhou, China; 3 Department of Radiation Oncology, Zhejiang Cancer Hospital, Hangzhou, Zhejiang, China

**Keywords:** Non-small cell lung cancer, Stereotactic ablative radiotherapy, Radiation-induced lung injuries, Progression free survival., Progression Free Survival, Ground Glass Opacities

## Abstract

**Objective:**

This study aimed to determine the relationship between Radiation-Induced Lung Injury (RILI) and the clinical outcome of Non-Small Cell Lung Cancer (NSCLC) following Stereotactic Ablative Radiotherapy (SABR).

**Methods:**

Clinical data and follow-up CT scanning of 101 patients with early NSCLC who received SABR treatment from January 2012 to December 2018 were retrospectively collected, and the Progression Free Survival (PFS) was calculated. CT features of peritumoral RILI were observed by 3 radiologists, each with 10 to 15 years of experience, based on consensus among 3 radiologists and divided into 3 types. Type 1: Diffuse consolidation surrounding the tumor, including the tumor boundary. Type 2: Ground Glass Opacities (GGOs) covering more than 180 degrees around the tumor. Type 3: GGOs surrounding the tumor but covering less than 180 degrees. Log-rank test was used to analyze the correlation between the classification of radiation-induced lung injury CT findings and PFS. Independent predictors of PFS rate were analyzed by COX multivariate regression.

**Results:**

The 5-year PFS rates based on RILI types observed at 6-8 months post-SABR were: Type 1 = 69.5%, Type 2 = 50.9%, and Type 3 = 36.1%. A statistically significant difference was observed among the three RILI types (p=0.025). COX multivariate regression analysis showed that RILI were independent factors influencing PFS (at 6-8 months follow-up after radiotherapy (p=0.041).

**Conclusion:**

Patients with more extensive and denser RILI tend to have a longer PFS. Data from our cohort study indicate that the 6- 8 months post-SABR period represents the optimal follow-up window, as evidenced by significant progression-free survival rate dynamics during this interval (HR = 1.5, 95% CI 1.0-2.2, p < 0.05).

## 1 INTRODUCTION

Lung cancer has the second highest incidence among malignancies and is the leading cause of cancer-related deaths worldwide [1]. Non-small cell lung cancer (NSCLC) accounts for 80%-85% of all lung cancers, and surgery remains the standard treatment for NSCLC [2]. However, Stereotactic Ablative Radiotherapy (SABR) is increasingly used to treat early-stage NSCLC patients who are unable to undergo surgery, and SABR has been established as a standard alternative therapy [3-5]. Previous studies [6-8] have shown that after SABR, the rate of local control for early NSCLC can reach 85% to 98%, and the 3-year overall survival rate can reach 48% to 65%. However, there is still a risk of local recurrence (4% to 14%) and distant metastasis (13% to 23%) in patients after SABR, which presents a challenge for clinicians [9, 10]. Therefore, it is important to identify patients with a high risk of disease progression. It is of great significance to establish an effective prediction model to evaluate the progression and survival probability of patients with early NSCLC for the selection of treatment schemes or individualized follow-up design.

Our previous study [11] showed that Radiation-Induced Lung Injury (RILI) was the only independent risk factor for early NSCLC progression after SABR. The manifestations of peritumor RILI on CT after SABR changed with time, and the change pattern of lung parenchyma could be divided into acute stage (corresponding to pneumonia within 6 months) or advanced stage (corresponding to fibrosis after 6 months) [12]. Determining the CT findings of peritumoral RILI at different time points and the specific association with tumor progression will help clinicians develop a reliable follow-up plan. This study will build on previous research to further analyze the relationship between RILI around tumors, disease surveillance, and disease progression.

## 2 MATERIALS AND METHODS

This retrospective study was conducted in accordance with the Declaration of Helsinki and was approved by the Ethics Committee of Zhejiang Cancer Hospital (Approval No. IRB-2022-394, June 25, 2022). All methods were carried out in accordance with relevant guidelines and regulations.

### 2.1 Study Population

Clinical and imaging data were collected for 101 early NSCLC patients who received SABR treatment in the thoracic radiotherapy department between January 2012 and December 2018. General information (such as gender, age, KPS, Carlson score, and smoking history), lesion status before treatment (such as diameter, location, pathological type, and T stage), radiation parameters (biologically effective dose, BED), the lung lobe involved, and CT classification of the adjacent RILI were collected. Inclusion criteria were: 1) primary NSCLC confirmed by bronchoscopy or percutaneous lung CT-guided biopsy, 2) clinical TNM stage T1-3N0M0 according to the American Joint Committee on Cancer (AJCC) 8th edition, 3) no prior anti-tumor therapy, and 4) chest CT follow-up every 3-6 months before and within 3 months after SABR. Exclusion criteria were: 1) concomitant primary malignancies, 2) incomplete clinical information and follow-up imaging data, and 3) loss to follow-up. Finally, 86 patients were included as study subjects.

### 2.2 SABR Treatment

All patients underwent a four-dimensional CT simulation. Lesions in the upper lobe of the lung were scanned by free breathing. In the lower lobe of the lung, abdominal compression plates were used to control large respiratory movements. The Internal Gross Tumor Volume (IGTV) originated from the Maximum Intensity Projection (MIP) of 4DCT, and the Planning Target Volume (PTV) was expanded by 5 mm in all directions around the IGTV. The total radiation dose and fraction dose were determined by a radiation oncologist based on the lesion location, volume, and surrounding organs at risk. The therapeutic dose of central lung cancer was 5-8Gy/F, with 8-10 times of irradiation. The therapeutic dose of peripheral lung cancer was 10-11Gy/F, and the radiation was divided into 4-6 times. The dose outside the 2cm range of PTV will be approximately half the prescribed dose. Target contouring, consistency, and dose constraints in normal tissues were based on the Radiation Therapy Oncology Group (RTOG) 0236 study [13]. The Biologically Effective Dose (BED) was calculated using the formula BED= nd (1+ d/α/β), where n is the number of fractions, d is the dose per fraction, and α/β is 10 Gy for lung cancer.

### 2.3 Follow-Up

All patients underwent CT examination within three time periods after treatment: <3 months, 3-5 months, and 6-8 months. If disease progression was suspected during follow-up, PET/CT or biopsy was performed. The endpoint of this study was tumor recurrence, and Progression-Free Survival (PFS) was calculated.

### 2.4 Imaging Acquisition Protocol

For chest CT scanning, a GE Definition Flash CT or Siemens SOMATOM Perspective CT was used with a tube current of 100-300mAs and tube voltage of 120kV. The slice spacing and thickness were 5.0mm.

### 2.5 Image Analysis

First, two experienced radiologists(FW and HY) with over 10 years of work experience classified peritumoral RILI in a double-blind manner. If there was disagreement, a third radiologist(HT) with over 15 years of experience in heart and lung diagnosis made the judgment. Peritumoral RILI was classified based on the range and density of radiation-induced lung injuries: Type 1: Diffuse consolidation surrounding the tumor, including the tumor boundary. Type 2: Ground glass opacities (GGOs) covering more than 180 degrees around the tumor. Type 3: GGOs surrounding the tumor but covering less than 180 degrees.

### 2.6 Statistical Analysis

All data were analyzed using IBM SPSS 24.0 (IBM Corp., Armonk, NY, USA). Independent sample t-tests were used for continuous variables that followed a normal distribution. Otherwise, the Wilcoxon rank-sum test was used to analyze continuous variables. The chi-square test or Fisher's exact test was used for categorical variables. A p-value less than 0.05 was considered statistically significant. The log-rank test was used to compare survival rates between two or more groups. The COX multivariate analysis was used to identify independent factors affecting PFS.

## 3 RESULTS

### 3.1 Univariate And Multivariate Analysis of Factors Affecting PFS

86 cases were included, of which 62 were male, and 24 were female, with a median age of 74 (51-88) years. There was no statistically significant difference between the clinical and pathological factors of patients and PFS. However, there was a remarkably statistically significant difference in PFS between patients with follow-up periods of 3-5 months and 6-8 months (Table **1**).

### 3.2 COX Multivariate Analysis of Factors Affecting PFS

The COX multivariate regression analysis was used to identify factors affecting PFS using clinical and pathological factors, radiotherapy, and CT features of RILI as variables. The CT features of peritumoral RILI at 6-8 months after radiotherapy were found to be an independent factor affecting PFS (Table **2**).

### 3.3 CT Features of Peritumoral RILI at Different Follow-up Times

**Fig. (1):**
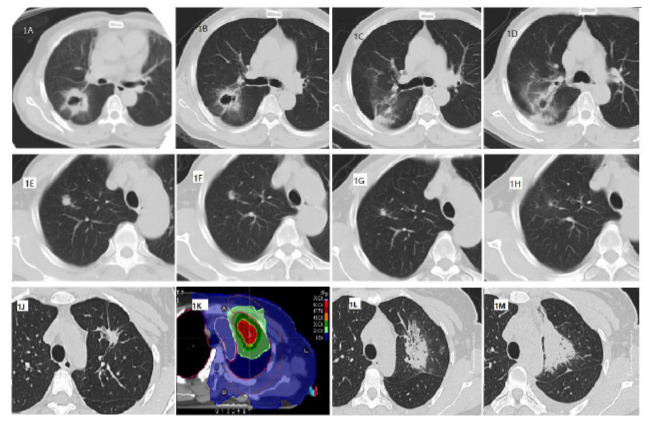
Classification of the types of peritumoral radiation-induced lung injuries A-D: A 70-year-old male patient, Puncture: non-small cell carcinoma with necrosis, T2bN0M0, PFS=82.3 months, A: before SABR, B: 0.7 months after treatment, peritumoral RILI Type3, C: 3.7 months after treatment, Type2, D: 6.7 months after treatment, Type2, no progress by the end of follow-up. E-H, another non-small cell carcinoma patient, male, 75 years old, T1bN0M0, PFS=22.5 months, E: before SABR, right upper lobe nodule, mildly lobulated, F: 1.5 months after treatment, peritumoral RILI Type3, G: 3.7 months after treatment, Type 3, H: 6.9 months after treatment, Type 2, PFS=22.5 months. J-M: A third NSCLC patient, female, 32 years old, with adenocarcinoma confirmed by puncture biopsy, PFS = 86 months. J: SABR target area plan diagram, DT50Gy/5F, L: 3 months and 10 days after treatment, with ground-glass opacity involving approximately 180 degrees of the peritumoral lung lobe (Type 2), M: 7 months after treatment, demonstrating increased parenchymal density, with the tumor obscured by consolidation (Type 1).

After SABR, the proportion of peritumoral RILI for types 1, 2, and 3 varied at different follow-up times (Fig. **1** and Table **3**).

### 3.4 Correlation Between Radiation-Induced Lung Injuries and PFS

**Fig. (2):**
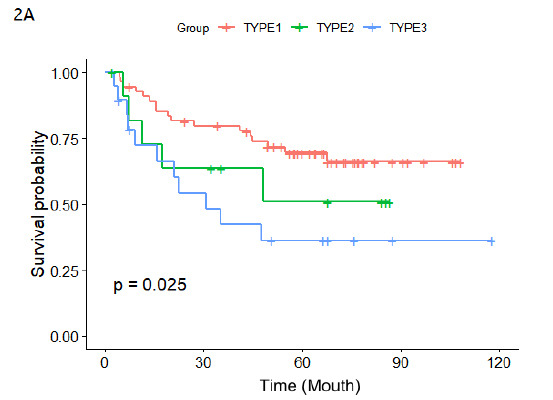
Progression-Free Survival (PFS) rates corresponding to three types of Radiation-Induced Lung Injury (RILI) observed at different time points after Stereotactic Ablative Radiotherapy (SABR) for early-stage Non-Small Cell Lung Cancer (NSCLC). 2A: KM curve of PFS for different RILI types, showing a statistically significant difference (p=0.025).

The CT features of RILI were not statistically related to the 5-year PFS rate within 3 months after SABR. The CT features of RILI at 3-5 months or 6-8 months after SABR had a statistically significant correlation with the 5-year PFS rate. In addition, survival curves demonstrated the effect of three radiation-induced lung injury CT types on disease progression at 6-8 months follow-up after SABR in early non-small cell lung cancer (Fig. **2** and Table **4**).

Compared with CT features of RILI at 1-2 months follow-up and 6-8 months follow-up, the classification of peritumoral radiation pneumonia changed with increasing time. Moreover, 57 patients demonstrated type 3 lesions at 1-2 months follow-up, 18 patients remained unchanged at 6-8 months follow-up, 6 patients developed type 2, and 33 patients developed type 1. Of the 23 patients who showed type 2 at 1-2 months follow-up, one patient developed a type 3 lesion at 6-8 months follow-up, 6 remained at type 2, and 16 patients developed type 1 lesions. All six patients who presented with type 1 at 1-2 months follow-up remained type 1 at 6-8 months follow-up.

### 3.5 PFS Analysis Results

At the end of the follow-up in December 2022, the follow-up time ranged from 6 months to 6 years, with a median time of 59.1 months. Disease progression occurred in 38.4% of the 86 patients. The 1-, 3-, and 5-year PFS rates were 84.4%, 69.6%, and 60.1%, respectively.

## 4 DISCUSSION

The role of SABR in the treatment of early non-small cell lung cancer has been demonstrated. In clinical practice, RILI associated with SABR is unavoidable. In this study, we established a comprehensive classification mechanism for RILI and divided the follow-up time into three distinct time periods. This study showed that the CT findings of RILI were statistically correlated with the PFS rate at 3-5 months and 6-8 months of follow-up. COX multivariate analysis showed that the lung injury CT features at 6-8 months after SABR follow-up were independent factors affecting PFS rate. Within the first 3 months and 6-8 months after SABR, the CT features of peritumoral radiation-induced lung injuries of patients also changed with an extension in follow-up time, and the type and proportion of the CT features of peritumoral radiation-induced lung injuries also changed. The proportion of patients with type 1 RILI CT features increased, while the proportion of patients with type 2 and type 3 lung injury decreased. The number of patients with CT features of type 1 peritumoral radiation-induced lung injuries reached 55 at 6-8 months after SABR, and the 5-year PFS rate was 69.5%. The number of other types of CT features of radiation-induced lung injury around tumor decreased, and the 5-year PFS rate of patients with type 2 CT and type 3 CT were 50.9% and 36.1%, respectively. The results showed that the follow-up at 6-8 months after SABR could reveal the early major CT features of radiation-induced lung injuries, and the classification of CT features at this time was more accurate and reliable in predicting the risk of recurrence, offering valuable guidance to follow-up planning.

Yang *et al*. [14] focused on RILI around the tumor after SABR in their work and believed that the CT findings of RILI were correlated with the prognosis of lung cancer after SABR through preliminary studies. The results showed that one month after SABR, more than half of the patients did not have peritumor lung injury on CT. The OS and PFS of these patients were significantly longer than those in patients with realistic changes and ground glass shadows around the tumor. This study has a short follow-up time after treatment, which cannot fully reflect the development and change process of radiation lung injury and needs further study. The average follow-up time in this study was ~59 months, which is longer than that of competing studies.

According to the range of peritumoral changes, this study divided the changes into three types. The CT findings of radiation lung injury in this study were basically consistent with those reported in the literature [15].

SABR treatment exhibits a small dose distribution range and high tumor conformality, usually only causing radiation changes in the high-dose area, and the radiation changes were limited to the region directly around the tumor. Literature has revealed that there are two mechanisms of radiation lung injury: DNA damage and reactive oxygen species production [16]. After mitochondrial DNA damage, it can cause inflammation, immune response, and cell apoptosis, which is closely related to immune-related lung diseases. The same view has also been seen in other literature [17], where radiation to tumors and surrounding lung tissue sets off a cascade of DNA damage and triggers the process of DNA damage repair. If the repair fails after injury, the cells die, and when the cells die, they attract immune cells, which then release pro-inflammatory cells that attract more immune cells to different areas of the lung. According to this mechanism, we believe that the more the tumor area and peritumoral cell death, the more obvious the local inflammation and immune response, the more inflammatory and immune cell aggregation, and the higher the density of lung tissue. Radiation-induced lung injury type I CT showed the highest density, indicating that there were more inflammatory and immune cells in the local area, and this immune environment may have a stronger killing and inhibiting effect on tumors, so patients with type I manifestations have better PFS.

A preclinical model on radiation-induced lung injury after SABR was conducted in mice [18]. The study found that SABR induced a heterogeneous group of senescent cells and identified Type II lung cells, macrophages, and endothelial cells as types of senescent cells, which appeared around the tumor 4-16 months after SABR and promoted both acute and late-phase lung toxicity. This animal model confirms that senescent cell populations occur in peritumor and induce inflammatory and immune responses after death, promoting the aggregation of inflammatory and immune cells into peritumor.

The retrospective nature of this study precluded the integration of RILI spatial mapping with SABR dosimetric data. Specifically, we were unable to perform dose-volume histogram analyses across distinct RILI severity grades and to co-register post-SABR CT images exhibiting RILI with pretreatment radiation plans to quantify dose gradients within injury zones. Future prospective studies incorporating advanced image fusion techniques are warranted to validate this hypothesis. The sample size of this study is relatively small,and this study is a single-center study and is limited to patient populations from specific regions or institutions, which may result in a lack of sample diversity. Future research should incorporate multi-center data to enhance the diversity and representativeness of the sample.

## CONCLUSION

Radiation-Induced Lung Injury (RILI) following Stereotactic Ablative Radiotherapy (SABR) may serve as a potential predictor for recurrence in early-stage Non-Small Cell Lung Cancer (NSCLC). Patients with more extensive RILI around the tumor demonstrated higher 5-year progression survival -free (PFS) rates compared to those with less extensive RILI. Additionally, some patients exhibited progressive worsening of CT features over time. Based on these findings, this study recommends using a radiographic classification of RILI 6-8 months post-SABR to assess recurrence risk and suggests considering additional local treatment for patients at high risk of recurrence. These conclusions are supported by the observed data, though further research is needed to validate these findings and refine clinical applications.

## Figures and Tables

**Table 1:** Univariate analysis showed the effects of clinicopathology, radiotherapy, and CT features of radiation pneumonia on PFS.

**Clinicopathological Factors**	**Number of Patients**	**Univariable Analysis**	
**Log-rank χ2**	***P* value**
**Age**			
<70y	32	0.037	0.847
>70y	54		
**Gender**			
0	24	0.275	0.6
1	62		
**KPS**			
<=80	16	0.126	0.723
>80	69		
**Carlson**			
0	46	0.612	0.736
1	22		
2~4	18		
**Smoking**			
No	35	0.544	0.461
Yes	51		
**T stage**			
1	64	1.321	0.517
2	21		
3	1		
**Tumor diameter**			
<=2cm	36	0.105	0.949
2-3cm	38		
>3cm	12		
**Tumor location**			
Central	5	1.676	0.195
Peripheral	81		
**Histology**			
Adenocarcinoma	43	1.308	0.52
Squamous cell carcinoma	26		
Otherwise	17		
**Involved lobe**			
RL	51	0.148	0.7
LL	35		
**BED>=100**			
NO	33	0.105	0.745
YES	53		
**Type of peritumoral RILI**			
**Follow-up <3 months**			
1	6	1.19	0.551
2	23		
3	57		
**Follow-up 3-5 months**			
1	40	10.338	0.006
2	21		
3	25		
**Follow-up 6-8 months**			
1 2 3	55 12 19	7.414	0.025

**Table 2:** COX multivariate analysis showed the effects of Follow-up time on PFS.

**Clinicopathological Factors**	B	SE	Wald	df	P value	Exp(B)	95.0%Cl	
downside	upside
**Follow-up 3-5 months**								
1	0.331	0.174	3.631	1	0.179	1.424	0.851	2.383
2								
3								
**Follow-up 6-8 months**								
1	0.468	0.080	33.868	1	0.041	1.507	1.017	2.232
2								
3								

**Table 3:** CT features of peritumoral radiation-induced lung injuries at different follow-up times.

**Type of Peritumoral RILI**	**Number of Cases (percentage)**		
**Follow-up <3 months**	**Follow-up3-5 months**	**Follow-up6-8 months**
1	6(7.0%)	40 (46.5%)	55(64.0%)
2	23 (26.7%)	21 (24.4%)	12(14.0)
3	57 (66.3%)	25(29.1%)	19(22.0%)

**Table 4:** CT features of radiation pneumonia were correlated with PFS.

Type of Peritumoral RILI	Number of Cases	PFS (%)			Log-rank χ^2^	*P*
1year	3years	5years
Follow-up <3 months						
1	6	83.3	83.3	83.3	1.190	0.551
2	23	86.4	72.7	63.0		
3	57	83.8	67.1	57.1		
Follow-up 3-5 months						
1	40	89.9	77.1	68.1	10.338	0.006
2	21	90.0	90.0	73.1		
3	25	70.6	39.7	35.3		
Follow-up 6-8 months					7.414	0.025
1	55	90.8	79.7	69.5		
2	12	72.7	63.6	50.9		
3	19	72.3	42.2	36.1		

## Data Availability

The data of current study are available from corresponding author [H.J], on a reasonable request.

## LIST OF ABBREVIATIONS

SABR: Stereotactic Ablative Radiotherapy
RILI: Radiation-Induced Lung Injury
PFS: Progression Free Survival
NSCLC: Non-Small Cell Lung Cancer
BED: Biologically Effective Dose

## STANDARDS OF REPORTING

STROBE guidelines were followed.
